# The potential mechanism of gut microbiota-microbial metabolites-mitochondrial axis in progression of diabetic kidney disease

**DOI:** 10.1186/s10020-023-00745-z

**Published:** 2023-10-31

**Authors:** Leilei Ma, Li Zhang, Jing Li, Xiaotian Zhang, Yiran Xie, Xiaochen Li, Bo Yang, Hongtao Yang

**Affiliations:** Present Address: Department of Nephrology, First Teaching Hospital of Tianjin University of Traditional Chinese Medicine, National Clinical Research Center for Chinese, Medicine Acupuncture and Moxibustion, Tianjin, 300380 China

**Keywords:** Gut microbiota, Microbial metabolites, Mitochondrial, Diabetic kidney disease

## Abstract

Diabetic kidney disease (DKD), has become the main cause of end-stage renal disease (ESRD) worldwide. Lately, it has been shown that the onset and advancement of DKD are linked to imbalances of gut microbiota and the abnormal generation of microbial metabolites. Similarly, a body of recent evidence revealed that biological alterations of mitochondria ranging from mitochondrial dysfunction and morphology can also exert significant effects on the occurrence of DKD. Based on the prevailing theory of endosymbiosis, it is believed that human mitochondria originated from microorganisms and share comparable biological characteristics with the microbiota found in the gut. Recent research has shown a strong correlation between the gut microbiome and mitochondrial function in the occurrence and development of metabolic disorders. The gut microbiome’s metabolites may play a vital role in this communication. However, the relationship between the gut microbiome and mitochondrial function in the development of DKD is not yet fully understood, and the role of microbial metabolites is still unclear. Recent studies are highlighted in this review to examine the possible mechanism of the gut microbiota-microbial metabolites-mitochondrial axis in the progression of DKD and the new therapeutic approaches for preventing or reducing DKD based on this biological axis in the future.

## Introduction

The prevalence of diabetic patients has been rising in recent decades, leading to an increase in cases of DKD, a severe microvascular complication of diabetes mellitus (DM) characterized by thickening of the basement membrane, glomerular and tubular hypertrophy, mesangial matrix expansion, and eventual loss of kidney function (Hoshino et al. 2015; Ritz, and Orth 1999). The International Diabetes Federation has released data predicting that the number of individuals with diabetes will reach 700 million by 2045 (Saeedi et al. 2019). After an initial diagnosis, approximately 40% of diabetic patients may develop DKD within 10–20 years (Lim 2014). DKD accounts for 44.5% of newly diagnosed ESRD patients and has become the main cause of ESRD (Collins et al. 2011; Sharma et al. 2017). As kidney damage progresses, DKD patients have a higher risk of mortality and decreased quality of life (Afkarian et al. 2013). Despite the impact of this, the current comprehension of the pathogenic mechanisms of DKD remains intricate and ambiguous. Currently, although more and more trials showed that the SGLT2 inhibitors have certain renoprotection (Garofalo et al. 2019; Mosenzon et al. 2019; Leslie and Gerwin 2019; Heerspink et al. 2020), effective treatments for DKD are still deficient (Hostetter 2001). Therefore, new prospective treatment strategies for DKD need to be explored urgently.

Accumulating evidence has emphasized a significant association between the microbiome of the digestive system and metabolic disorders like obesity, polycystic ovary syndrome, and diabetes (Tilg, and Moschen 2014; Wu et al. 2021). Emerging research suggests that an imbalance in gut bacteria can impact the advancement of CKD by generating uremic toxins and controlling inflammation and the immune system (Lee et al. 2011). Several studies have shown that the alteration of gut microbiota play a crucial part in the onset and development of DKD (Vaziri et al. 2016; Fernandes et al. 2019). However, a key question is to answer how gut microbiota results in renal damage in DM patients. As we know, gut microbiota dysbiosis is characterized by a disruption of the homeostatic balance and abnormal production of bacterial metabolites. Although some gut microbiota metabolites, which can be either beneficial or harmful, were found participated in the pathological progression of DKD (Fang et al. 2021a), there are still numerous challenges to uncover the underlying pathological mechanism.

The fact that the renal system has a high resting metabolic rate is well-known, which leads to an abundance of mitochondria. High energy produced by mitochondria must meet the high energy requirements of the kidney for glomerular filtration, urine reabsorption and formation (Bhargava, and Schnellmann 2017). To maintain a healthy and optimally functioning kidney, it is crucial to have mitochondrial homeostasis as kidney cells like proximal tubular cells and podocytes require adenosine triphosphate (ATP) and reactive oxygen species (ROS) that are released by mitochondria to perform their physiological functions (Müller-Deile and Schiffer 2014; Gilbert 2017; Brinkkoetter et al. 2019). Extensive research has been conducted in recent years on the possible contribution of mitochondrial malfunction to the onset and advancement of DKD. Sufficient research indicates that changes in mitochondria, such as their shape, creation, energy production, and excessive production of ROS, play a role in the advancement of DKD (Bhargava and Schnellmann 2017; Galvan et al. 2017, 2021).

Considering the comprehensive crosstalk between the microbiome and mitochondria is an intriguing perspective, as previously stated, due to the connection between dysbiosis of gut microbiota and changes in mitochondria in the pathogenesis of DKD. Host mitochondria can import certain bacterial proteins because their targeting sequences are similar to those of mitochondria (Lucattini et al. 2004). The biological processes of mitochondria (Franco-Obregón and Gilbert 2017; Lobet et al. 2015; Neish, and Jones 2014) can be directly regulated by the gut microbiota through key transcription factors, coactivators, and enzymes. Our hypothesis is that communication between mitochondria and the human microbiome can impact the metabolic health of the host. It is noteworthy that multiple researches highlight the dependability of the inter-action between the microbiome and mitochondria of the host (Lobet et al. 2015; Walker et al. 2014; Zorov et al. 2014). A significant role in communication between bacteria and mitochondria is thought to be played by metabolite molecules from gut microbiota, including short-chain fatty acids (SCFA), lipopolysaccharides (LPS), and hydrogen sulfide (H2S) (Mafra et al. 2019). Therefore, this article aims to investigate the possible mechanism of the gut microbiota-microbial metabolites-mitochondrial axis in the advancement of DKD by analyzing existing literature and propose potential therapeutic strategies for managing DKD.

## Gut microbiota dysbiosis in DM and DKD

### Alteration of gut microbiome in type 1 and 2 DM individuals

While a direct link between gut microbiota dysbiosis and T1DM development has not been confirmed, an imbalance in gut microbiota has been found at different stages of diabetes, which includes T1 and T2 DM. In patients with prediabetes or diabetes, the gut microbiota changes characterized by a decrease in the ratio of Gram-positive firmicutes to Gram-negative Bacteroidetes (Demirci et al. 2020; Giongo et al. 2011), absence of butyrate producing bacteria, reduced bacterial diversity and community stability, which is significantly distinct from that of healthy individuals (Knip, and Siljander 2016; Brown et al. 2011; De Goffau et al. 2013; Kostic et al. 2015). Bacteroides are associated with increased interleukin (IL)-6, poor blood glucose control, increased toll-like receptor levels and anti-islet cell autoantibodies (Demirci et al. 2020; Higuchi et al. 2018; Huang et al. 2018) in T1DM patients. Decreased Faecalibacterium levels was also observed in MODY2 patients, which is negatively related with HbA1c (Huang et al. 2018). Under germ-free (GF) conditions, the persistent deterioration of insulitis in T1DM patients suggests a possible signaling crosstalk between the immune system and the microbiota (Alam et al. 2011). Autoimmunity of β cell in islet has been shown to be associated with changes in specific symbiotic bacteria, including reduction of clostridium leptin in non-obese diabetic rats and increase of bacteroidetes in late T1DM patients (Sysi-Aho et al. 2011; Davis-Richardson et al. 2014).

New research in both humans and animals has revealed that the gut microbiota of individuals with T2DM has undergone significant alterations. In T2DM patients, the hyperglycemia and metabolic disorder were demonstrated to be positively correlated with a higher ratio of Firmicutes/Bacteroidetes (Remely et al. 2016; Wu et al. 2019).

Compared to healthy controls, T2DM patients exhibit a greater percentage of Enterobacteriaceae, Collinsella, Streptococcus, Lactobacillus and Lachnospiraceae/Ruminococcus, which was important in activating lower levels of inflammation and exacerbating insulin resistance (Candela et al. 2016). In addition, the probiotics that produce SCFAs including the bacteroidesis prevotellais lachnospirag roseburia and faecali bacteria were significantly depleted in patients with diabetic (Ma et al. 2020). Compared with healthy subjects, the abundance of Blautia, a producer of SCFAs, is reduced in T2DM patients, which was like some other butyric acid-producing bacteria and may be one of the causes of dysglycemia (Song et al. 2020; Inoue et al. 2017). To sum up, current evidence shows that altered gut microbiome are significantly related to the pathogenesis of type 1 DM or type 2 DM (Vatanen et al. 2016; Brown et al. 2016). Altered gut microbiome may promote the onset of type 1 diabetes by changing the immune system. In addition, Dysbiosis of gut microbiome may cause blood glucose regulation disorder by aggravating oxidative stress damage, promoting the expression of pro-inflammatory factors and increasing insulin resistance, which contribute to the development of metabolic disorders such as pre-diabetes and type 2 diabetes (Palacios et al. 2017; Peng et al. 2014).

### Changes of gut microbiota in DKD

Abnormal gut microbiota has been observed in both animal and human studies about DKD. The DKD animal model exhibited a reduction in α-diversity and SCFAs producing bacteria, along with an increase in Bacillota and Actinobacteria (Li et al. 2020b). In the deterioration of DKD, the metabolic disorders were mainly caused by g_Eubacterium_nodatum_group, g_Lactobacillus, and g_Faecalibaculum as concluded by Zhang et al. (2022). A study in rats explored the pathophysiological mechanism between early DKD and changes in gut microbiota. The findings indicated that DKD rats, induced by intraperitoneal injection of streptozotocin, exhibited abnormal gut microbiota and an elevated level of plasma acetate. Elevated acetate levels hastened the onset of renal damage, including thickening of the glomerular basement membrane, fusion of podocyte feet, and hypertrophy of the mesangial matrix (Lu et al. 2020). The abundance of certain bacteria is associated with the severity of proteinuria. In DKD mice model with severe proteinuria (24 h urinary protein ≥ 300 mg/24 h), the enrichment of Allobaculum and Lottis was significantly increased, and was positively correlated with body weight, blood glucose and 24 h urinary protein, while Blautia was negatively correlated with 24 h urinary protein in the mild proteinuria group (24 h urine protein < 300 mg/24 h) (Li et al. 2020a).

He et al. discovered that the gut microbiota of DKD patients differed significantly from T2DM patients, and that the abundance of certain Citrobacter farmeri and Syndromus schinkii was positively associated with the the urinary albumin/urine creatinine ratios (UACRs) of DKD patients (He et al. 2022). A study from China analyzed the variation in gut microbiota among healthy individuals, DKD patients who were diagnosed by renal biopsy and T2DM patients without kidney injury. The result exhibited notable variations in the abundance and variety of intestinal microorganisms among patients with DKD and T2DM. Compared to T2DM patients, DKD group had a higher abundance of Proteobacteria and Eschericha-Shigella, but a lower abundance of Prevotella_9 (Tao et al. 2019). Firmicutes, which produce butyrate and regulate the inflammatory response, were found to be more abundant in Control and T2DM groups than in DKD patients (Wong et al. 2014; Furusawa et al. 2013). Du et al. (2021) observed dysbiosis and reduced richness and diversity of gut bacteria from phylum to genus levels in DKD patients, which could potentially serve as new microbial biomarkers for DKD. In addition, a systematic review and meta-analysis found that DKD patients had lower levels of intestinal bacteria, decreased diversity index, and significant changes in β diversity compared to healthy controls. The most significant changes were an increase in *Escherichia*, *Citrobacter*, and *Klebsiella*, and a reduction in Roseburia (Wang et al. 2022).

To summarize, although an intimate relationship between alterations of composition and function in gut microbiota and the progression of DKD has been confirmed, it is essential to clarify the specific mechanism of intestinal flora promoting the onset and advancement of DKD. Gut microbiota metabolites, as important messengers of communication between bacteria and host, may have a significant impact on regulating host immunity and inflammation (Uchimura et al. 2018). In this regard, we speculate that the change of metabolites derived from gut microbial may be a major bridge to further understand the ‘inter-talk’ between gut microbiome and DKD.

## The potential mechanisms of gut microbial metabolites in DKD

Upon consumption of macronutrients, the microbiome in the human gut has the ability to generate a range of metabolites including SCFAs, TMAO, bile acids (BAs), protein-bound uremic toxins (PBUTs), branched-chain amino acids (BCAAs), and some other unknown metabolites. The metabolites derived from gut microbial are considered to be the medium of communication between microbes and the host, which have significant effects on the biological activities and metabolism of the human body (Schroeder and Bäckhed 2016). In recent years, an increasing number of studies have investigated the changes in the diversity and function of gut microbiota in patients with metabolic diseases such as diabetes, obesity, and metabolic syndrome. Studies have found that these patients have significant changes in the gut microbial community, leading to dysbiosis of gut microbiota and/or leaky gut syndrome, increased intestinal permeability, dysfunction of intestinal barrier. Subsequently, a variety of gut microbiota metabolites are released into the blood, such as SCFAs, TMAO, LPS, and uremic toxins, are released into the blood, which further causes changes in disease phenotypes through a variety of signaling pathways (Koppe et al. 2018; Sharma and Tripathi 2019; Sharma et al. 2019; Jaworska et al. 2022).

Increasing evidence supports the crucial role of gut microbiota metabolites in the pathogenesis of DKD (Table 1). The major metabolites of microbiota-mediated fermentation of non-digestible carbohydrates in the gut are SCFAs, which are primarily produced by Bacteroidetes and Firmicutes (Levy et al. 2016). Research has demonstrated that SCFAs have a significant impact on combatting inflammation, controlling immune response, and exerting anti-oxidant and anti-fibrotic effects in kidneys. Furthermore, SCFAs play a role in regulating blood pressure and human metabolism by activating G protein-coupled receptors and inhibiting histone acetylation (Huang et al. 2017b; Li et al. 2019, 2021). In comparison to T2DM individuals without kidney disease and normal controls, Zhong and colleagues found that patients with DKD had decreased levels of SCFAs in both their serum and feces. SCFAs are negatively correlated with renal function (Zhong et al. 2021). The pathological damage and deterioration of renal function in DKD can be improved by exogenous SCFAs, particularly butyric acid, through the inhibition of oxidative stress and NF-kB signaling mediated by GPR43 (Huang et al. 2020). Consuming dietary fiber can safeguard against DKD by promoting the growth of SCFA-producing microorganisms in the gut and elevating SCFA levels in fecal matter and blood serum, which can slow down the advancement of DKD by stimulating GPR43 and GPR109A receptors (Li et al. 2020b). Butyrate (NaBu) has been demonstrated to enhance renal pathological injury in diabetic rats induced by streptozocin(STZ) in vivo and alleviate apoptosis of NRK-52E cells induced by high glucose through inhibiting HDAC2 in vitro (Khan, and Jena 2014; Dong et al. 2017; Du et al. 2020). Conversely, there are disagreements regarding the function of acetate in DKD. Huang et al. Demonstrated that acetate has the ability to protect Mesangium cells against inflammation and oxidative harm caused by high glucose and Lipopolysaccharide (Huang et al. 2017a). While some research has indicated that acetate may have a negative impact on the progression of DKD. According to Lu et al.’s findings, the levels of plasma acetate was positive correlated with angiotensin II protein in the kidney, which is believed to be a potential cause of DKD (Li et al. 2020a). Additionally, another study indicated that acetate can lead to tubulointerstitial injury in DKD by disrupting cholesterol homeostasis through the activation of GPR43 (Hu et al. 2020).

**Table 1:** A summary of current studies about the impact of gut microbial metabolites in DKD in vivo and in vitro

Metabolites	Human/animal/cell type species	Conclusions	Reference and year
SCFAs (acetate, butyrate, propionate)	DKD patients	The levels of serum and fecal SCFAs (especially in fecal) are lowered, and which are negatively correlated with renal function	Zhong et al. (2021)
SCFAs (acetate, butyrate, propionate)	C57BL/6 mice, T2D Mouse model induced by HFD and STZ, glomerular mesangial cells	SCFAs, especially butyrate, improved T2D-induced kidney damages including reduction of proteinuria, serum creatinine, urea nitrogen, and cystatin C, inhibition of mesangial matrix accumulation and renal fibrosis via GPR43-mediated inhibition of oxidative stress and NF-κB signaling,	Huang et al. (2020)
High-fiber diet, SCFAs (acetate, butyrate, propionate)	C57BL/6, Gpr43−/− and Gpr109A−/− mice; Mouse kidney tubular epithelial cells and podocytes	SCFAs can ameliorate renal damages, inhibit the expression of fibrosis-related genes (TGF-β and fibronectin), and decrease the inflammation in renal tubular cells and podocytes exposed to hyperglycemic by activating GPR43 or GPR109A receptors	Li et al. (2020)
NaB	Juvenile Sprague Dawley rats	NaB treatment can improve the renal function and alleviate the pathological injury, fibrosis, apoptosis and DNA damage in the diabetic kidney	Khan et al. (2014)
NaB	C57BL/6(Nrf2+/+) and Nrf2−/− mice	NaB treatment protect against DN by activating Nrf2 possibly via inhibition of HDAC activity	DDong et al. (2017)
NaB	db/db mice, NRK-52E cells	Sodium butyrate plays an anti-apoptotic effect in the kidney of db/db mice and HG-induced NRK-52E cells by inhibiting expression of HDAC2	Du et al. (2020)
SCFAs (acetate, butyrate, propionate)	C57BL/6 mice, Mouse glomerular mesangial cells	SCFAs, especially acetate and butyrate, significantly inhibit proliferation of GMCs,production of ROS and MDA, expression of ICAM-1 and proinflammatory cytokine(MCP-1and IL-1β) induced by high glucose and LPS	Huang et al. (2017a, b)
TMAO	Sprague Dawley rats	TMAO treatment not only aggravates the renal dysfunction and fibrosis, but also accelerates renal inflammation by activating NLRP3 inflammasome and releasing IL-1β and IL-18	Fang et al.(2021a, b)
Bile acids (FXR/TGR5 Dual Agonist)	DBA/2 J mice, C57BL/6 J mice, db/db mice	INT-767, as a semisynthetic bile acid derivative, can reduce proteinuria and relieve podocyte injury, mesangial expansion, and tubulointerstitial fibrosis via multiple pathways and targets	Wang et al. (2018)
UDCA	db/db mice, podocyte	UDCA exerts renoprotective effects by reducing the occurrence of oxidative stress and exhibits renal protection in vivo and in vitro	Cao et al.(2016a, b)
UDCA and 4-PBA	db/db mice, podocyte	UDCA Reduces renal pathological injury and apoptosis of podocytes by inhibiting activation of caspase-3 and caspase-12 and restoring autophagy in vivo and vitro	Cao et al. (2016a, b)
TUDCA	db/db mice, eNOS−/− mice, Podocytes, Human proximal tubular epithelial cells	TUDCA ameliorates tubular damage by inducing expression of FXR-dependent genes (SOCS3 and DDAH1) in tubular cells,db/db and eNOS−/− mice	Marquardt et al. (2017)
TUDCA	db/db mice	TUDCA reduces blood glucose, albuminuria and renal histopathology by inhibiting ER stress in the kidneys of diabetic db/db mice	Zhang et al. (2016)
IS	C57BL/6 mice, FVB/N mice, human kidney autopsy podocyte	IS promotes glomerular and podocyte injury including altered cell morphology, decreased expression of podocyte differentiation markers, and a proinflammatory state by activating podocyte AhR	Ichii et al. (2014)
IS	T2D patients	Level of serum IS is negatively correlated with renal function	Atoh et al. (2009)
PS	C57BL6 mice,db/db mice, KKAy mice, diabetes patients	The level of plasma PS is not only significantly correlated with proteinuria/creatinine and estimated glomerular filtration rate in diabetic patients, but also can predict the deterioration of ACR in DKD patients in 2 years	Kikuchi et al. (2019)

*SCFA* short-chain fatty acids; *HFD* high fat diet; *STZ* streptozocin; *GPR43* G-protein-coupled receptor 43; *NF-κB* nuclear factor kappa B; *GPR109A* G-protein-coupled receptor 109A; *TGF-β* transforming growth factor-β; *NaB* sodium butyrate; *HDAC* histone deacetylase; *NRF2* nuclear factor erythroid 2-related factor 2; *HG* high glucose; *NRK-52E* normal rat kidney tubular epithelial cells; *GMCs* glomerular mesangial cells; *ROS* reactive oxygen species; *MDA* malondialdehyde; *ICAM-1* intercellular cell adhesion molecule-1; *MCP-1* monocyte chemotactic protein-1; *IL-1β*interleukin-1 β; *LPS* lipopolysaccharide; *TMAO* Trimethylamine *N*-oxide; *NLRP3* nucleotide-binding domain, leucine-rich-containing family, pyrin domain-containing-3 inflammasome; *IL-18* interleukin-18; *FXR* farnesoid X receptor; *TGR5* G-protein-coupled BA receptor 1; *UDCA* Ursodeoxycholic acid; *4-PBA* 4-phenylbutyrate; *TUDCA* tauroursodeoxycholic acid; *eNOS* endothelial nitric oxide synthase; *SOCS3* suppressor of cytokine signaling 3; *DDAH1* dimethylarginine dimethylaminohydrolase 1; *IS* Indoxyl sulfate; *AhR* aryl-hydrocarbon receptor; *PS* phenyl sulfate; *ACR* albumin to creatinine ratio

The degradation of l-carnitine and choline found in foods like red meat, eggs, and cheese (Wang et al. 2011) results in the production of TMAO. Numerous researches have demonstrated that TMAO plays a role in controlling lipid metabolism and glucose balance, and is a factor in the development of some diseases such as atherosclerosis (Wang et al. 2011), heart failure (Tang et al. 2015b), diabetes (Zhuang et al. 2019), Alzheimer’s disease (Vogt et al. 2018), and chronic kidney disease (Tang et al. 2015a). A clinical Study have verified that T1DM individuals with higher levels of plasma TMAO are more likely to experience poor renal outcome (Winther et al. 2019). Increased levels of TMAO in the bloodstream are also linked to both cardiovascular disease events and mortality. TMAO can activate the NF-KB pathway in DKD patients, further aggravating the microinflammation in vivo and leading to DKD (Al-Obaide et al. 2017). DKD patients exhibited a higher concentration of TMAO in comparison to T2DM patients without kidney disease and healthy individuals, which was positively correlated with UACRs (Yang et al. 2022). In animal studies, DKD rats fed with TMAO showed more severe renal function decline and renal fibrosis. Further study has shown that TMAO can speed up kidney inflammation by activating the NLRP3 inflammasome and ultimately resulting in the discharge of IL-1β and IL-18 (Fang et al. 2021b).

The liver produces primary bile acids (BAs) which are later converted into secondary BAs by the gut microbiome (Matsubara et al. 2013). BAs regulates metabolism mainly by activating two primary receptors, namely the nuclear farnesoid X receptor (FXR) and the membrane-bound Takeda G protein-coupled receptor 5 (TGR5) (Chiang and Ferrell 2019), which have been demonstrated to exert renoprotective effects in diabetes and obesity (Wang et al. 2018). Ursodeoxycholic Acid (UDCA), one of the secondary BAs, has been discovered to alleviate renal dysfunction, podocyte apoptosis, and oxidative stress caused by renal ER stress in DKD rats (Cao et al. 2016a, b). Administering Tauroursodeoxycholic acid (TUDCA) can attenuate glomerular and tubular damage in diabetic rats, which is partly mediated by inhibiting ER. (Marquardt et al. 2017; Zhang et al. 2016).

PBUTs, including phenyl sulfate (PS), pcresyl sulfate (pCS), p-cresyl glucuronide (pCG) and indoxyl sulfate (IS), are generated by intestinal microorganism through the breakdown of aromatic amino acids and can’t be effectively eliminated by traditional hemodialysis method due to their protein-binding properties. Both in vitro and in vivo studies, indoxyl sulfate exposure caused renal tubulointerstitial and vascular injury and decreased expression of podocyte characteristic markers. Further studie showed that the above injury was induced by IS through activating aryl-hydrocarbon receptor (AhR) (Ichii et al. 2014). In DKD patients and rats (Van Der Kloet et al. 2012; Atoh et al. 2009; Zhao et al. 2012), there is a strong correlation between elevated intrarenal IS levels and 24-h urinary protein levels, estimated glomerular filtration rate (eGFR), and tubulointerstitial injury index. Another research has shown that elevated pCS levels are linked to the advancement of DKD (Niewczas et al. 2014). It has recently been found that the high level of plasma PS can cause reduction of mitochondrial function in podocyte, foot process disappearance, glomerular basement membrane (GBM) thickening and perivascular fibrosis, and the plasma PS level is not only significantly correlated with proteinuria/creatinine and eGFR in diabetic patients, but also can predict the deterioration of ACR in DKD patients in 2 years (Kikuchi et al. 2019). Therefore, PS can be used as a predictor for the risk of renal damage progression, a marker for early diagnosis, and a possible therapeutic target of DKD.

There is mounting evidence indicating that gut microbiota metabolites can serve as pathological and physiological characteristics or biomarkers of DKD, but there is still much uncertainty regarding this issue at present. Firstly, more research is needed to elucidate the causal relationship between microbial metabolites and DKD. Secondly, further exploration of the targets and receptors of these microbial metabolites in the human body is needed. Ultimately, we need to determine whether supplementation of beneficial microbial metabolites can alleviate or delay the progression of DKD.

## The interaction between gut microbial metabolites and mitochondria

Existing research has confirmed that there are some similarities in physiological characteristics and structure between gut microbiome and mitochondria, both of which jointly regulate host metabolism and longevity (Tomtheelnganbee et al. 2022; Ghosh et al. 2022). We hypothesize that there may be a potential cross-talk within the gut microbiome and the host mitochondria because existing studies have provided ample supporting evidence for this interaction mechanism. The origin of mitochondria can be traced back to methanogenic archaea in accordance with the prevailing endosymbiotic theory (Sagan 1967). Numerous studies have shown that rickettsia bacteria are ancestors of mitochondria. This origin theory indicates that mitochondria have similar biological properties with gut microbiota (Andersson et al. 1998; Fitzpatrick et al. 2006; Wang, and Wu 2015). Structurally and functionally, there are similarities as well. The inner membrane of mitochondria is similar to bacterial membrane, and the outer membrane is similar to eukaryotic biofilm. Mitochondrial DNA are similar to DNA from some bacteria (Wang, and Wu 2014). Mitochondria also share common autophagic systems as bacteria for membrane degradation (Degli Esposti et al. 2014). Recent research has started to uncover the intimate connection between the gut microbiota and mitochondria in the pathophysiology of different diseases (Gruber, and Kennedy 2017), while the gut microbiome metabolites may be a key medium responsible for this cross-talk.

Short-chain fatty acids (SCFAs) have the ability to hinder the inflammatory response and promote the advantageous outcomes of physical activity by stimulating important agents in mitochondrial biogenesis through transcriptional co-activators like peroxisome proliferator-activated receptor-γ coactivator-1α(PGC-1α), silent information regulator 1(SIRT1) and the enzyme AMPK (Clark, and Mach 2017). Butyrate can alter mitochondrial function, efficiency, and dynamics by improving respiratory capacity and fatty acid oxidation, activating the AMPK-acetyl-CoA carboxylase pathway, and promoting inefficient metabolism (Mollica et al. 2017). In a lymphoblastoid cell line isolated from boys with autism spectrum disorders, butyrate has the ability to improve mitochondrial function during physiological stress and/or mitochondrial dysfunction (Rose et al. 2018). PA levels in serum and stool are reduced in individuals with MS in comparison to controls. Positive outcomes were observed after three years of PA intake, such as a decrease in yearly relapse rate, stabilization of disability, and a reduction in brain atrophy, which were closely linked to the normalized Treg cell mitochondrial function and morphology in MS patients (Duscha et al. 2020).

Videja et al. showed that increased levels of TMAO can preserve fatty acid oxidation and reduce pyruvate metabolism, which can prevent monocrotaline-induced impairment of mitochondrial energy metabolism, despite the fact that high intake of TMAO and its precursor has been linked to worsening of atherosclerosis and CVD (Videja et al. 2021). Indole-3-propionic acids (IPA), which is mainly produced by Clostridium sporogenes, was reported to affect mitochondrial respiration in cardiomyocytes in an experimental model of right ventricular heart failure (Gesper et al. 2021). UDCA was considered to be a new approach for Parkinson's disease (PD) due to its ability to improve mitochondrial function and protect mitochondrial integrity (Abdelkader et al. 2016). The up-regulation of mitophagy by TUDCA in human neuroblastoma cells can prevent mitochondrial dysfunction and cell death, offering new perspectives for the prevention of neurodegenerative diseases (Fonseca et al. 2017). Delta-valerobetaine is an intestinal microbial metabolite that regulates the oxidation of mitochondrial fatty acids, which can result in increased lipid storage in adipose tissue and the liver, and further leading to obesity and hepatic steatosis (Liu et al. 2021). Administration of Urolithin A (UroA), a major metabolite of ellagic acid produced by the gut microbiome, can improve obesity and insulin resistance by attenuating triglyceride accumulation and elevating mitochondrial biogenesis in the liver (Toney et al. 2019). According to another research, UroA has the potential to trigger mitophagy, extend the lifespan of C. elegans, and enhance muscle performance in rodents (Ryu et al. 2016). Herpes simplex virus type 1 (HSV-1) can activate microglia by increasing mitochondrial damage through defective mitophagy, while the microbial metabolite NAMO can inhibit microglia activation and HSV-1 induced herpes simplex encephalitis (HSE) progress by restoring NAD+ dependent mitophagy (Li et al. 2022, 2023). Trimethyl-5-aminovaleric acid produced by gut microbiota can accelerate myocardial hypertrophy by altering mitochondrial ultrastructure, inhibiting carnitine metabolism and reducing fatty acid oxidation (Zhao et al. 2022). A harmful metabolite N6 carboxymethyl lysine (an advanced glycosylation end product) from the gut can cross the intestinal barrier and enter the body. It can damage microglia in the brain by increasing the reactive oxygen species (ROS), inhibiting mitochondrial activity and ATP accumulation. This suggests that enhancing the intestinal barrier function can reduce the translocation of harmful intestinal metabolites into the brain, which may be an effective means of promoting brain health in the elderly (Mossad et al. 2022). PS administration induces podocyte damage by decreasing mitochondrial basal respiration, ATP production, H+ leaking and maximum respiration capacity, which contributes to albuminuria and the progression of DKD (Kikuchi et al. 2019).

**Fig. 1:**
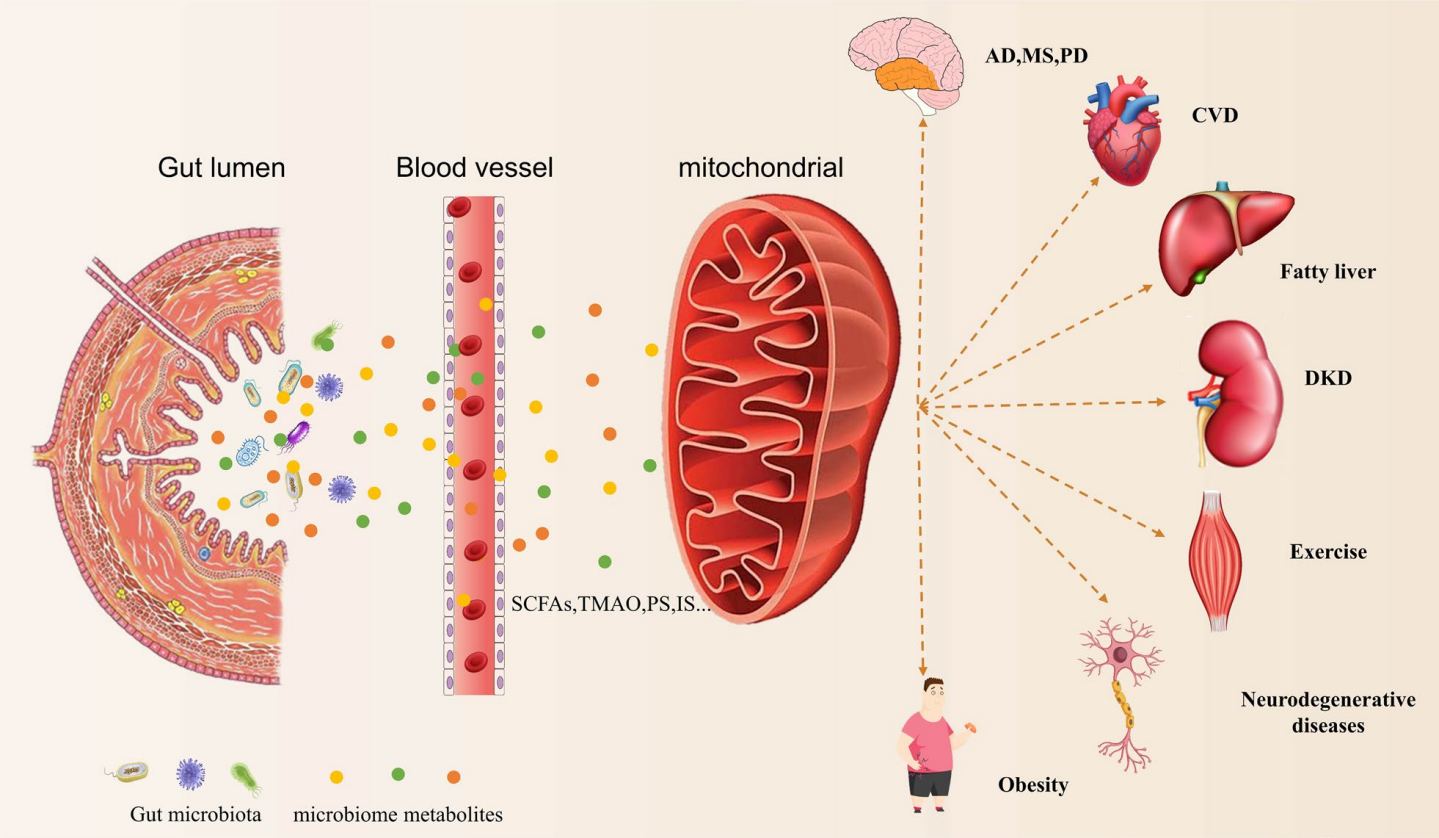
The interaction between metabolites produced by gut bacteria and mitochondria in chronic disease, as shown in this figure. With the imbalance of intestinal flora, the intestinal tract undergoes a number of structural and functional changes including leaky gut syndrome, increased intestinal permeability, dysfunction of intestinal barrier. Various microbial metabolites are released into the bloodstream. Many microbial metabolites were found to cause human diseases by affecting normal mitochondrial function. *SCFAs* short-chain fatty acids, *TMAO* trimethylamine *N*-oxide, *IS* indoxyl sulfate, *PS* phenyl sulfate, *AD* Alzheimer’s disease, *MS* multiple sclerosis, *PD* Parkinson’s disease, CVD cardiovascular diseases, *DKD* diabetic kidney disease

To summarize, the interaction between intestinal microorganisms and mitochondria is fascinating and associated with pathogenic state in different systemic diseases (Fig. 1). By targeting the dialogue between gut microbiota and mitochondria, a series of chronic diseases related to mitochondrial disorders and metabolic disorders can be more precisely treated (Franco-Obregón and Gilbert 2017). Gut microbiota strengthens its association with mitochondria by regulating the production of ROS and mitochondrial activity through the release of metabolites, proteins, or toxins. Establishing a chemical communication mode between bacteria and mitochondria is crucial for comprehending the intricate and ever-changing interactions between the environment, microbiome, and host, ultimately leading to a better understanding of their effects on health and diseases (Han et al. 2019). Such knowledge may allow clinician to treat mitochondrial and metabolic diseases by fine-tuning the quality and diversity of microbiota using treatment strategies such as probiotics administration, diet control or fecal transplantation. Such treatment strategy may avoid major research efforts required to demonstrate safety and effectiveness associated with direct application of large number of microbiota metabolites and microbiota related factors.

## Mitochondrial damage in DKD

Multiple pathological mechanisms are involved in the pathogenesis of DKD, including abnormal RAS system, excessive ROS production, increased AGEs, microinflammation, and oxidative stress. Nonetheless, an increasing amount of proof suggests that DKD’s pathogenesis is significantly influenced by mitochondrial impairment (Saxena et al. 2019; Wei, and Szeto 2019). Mitochondrial damages mainly include abnormal mitochondrial biosynthesis (Popov 2020), mitochondrial dynamic imbalance (Tur et al. 2020; Sabouny and Shutt 2020), mitochondrial dysfunction (Detmer and Chan 2007), and mitophagy disorder (Westermann 2010). The process of mitochondrial biosynthesis enables the nucleus and mitochondrial genome to work together closely in order to synthesize and replace damaged or dysfunctional mitochondria, which helps to maintain the stability of mitochondria and regulate their normal metabolic processes in cells (Weinberg 2011). Mitochondrial biosynthesis is regulated by PGC-1α, which plays a significant role in the development of DKD by enhancing mitochondrial production, revitalizing mitochondrial membrane potential, eliminating ROS, and preventing oxidative stress (Cai et al. 2016; Lu et al. 2017; Pettersson-Klein et al. 2018; Xue et al. 2019). Numerous researches indicate that the onset and progression of DKD (Guo et al. 2015; Lee et al. 2017, 2021; Lynch et al. 2018) are characterized by significant reduction in mitochondrial biogenesis and decreased expression of PGC1α. In animal models of DKD (Yuan et al. 2012), podocytes and mesangial cells can suffer damage due to decreased levels of PGC-1α and mitochondrial synthesis. However, some studies found that in a DKD mouse model, specific induction of podocyte overexpression of PGC1α failed to protect the kidney, instead causing podocyte damage and increasing urinary protein (Li et al. 2017). To conclude, DKD's pathogenesis is directly related to mitochondrial biosynthetic dysfunction.

Mitochondrial dynamics refers to the fact that mitochondria are in the Dynamic equilibrium of fusion and fission. This dynamic change of mitochondria can be manifested in morphological heterogeneity, such as punctate, fragmented, strip or linear in the cytoplasm. Changes in the cellular environment can affect mitochondrial fusion and fission by triggering changes in the function or activity of mitochondrial fusion or fission related proteins. Mitochondrial fission is mainly mediated by dynamic related protein 1 (DRP1), mitochondrial fission protein 1 (Fis1) and mitochondrial fission factor (MFF). The fusion process is divided into the fusion of outer mitochondrial membrane (OMM) and inner mitochondrial membrane (IMM), which are respectively mediated by mitochondrial fusion protein (MFN) and optic atrophy protein 1 (OPA1). Knockout of Drp1 can significantly reduce mitochondrial fission, decrease albuminuria and improve podocyte morphology, while the activation of Mfn1/2 can alleviate lesions of DKD (Audzeyenka et al. 2022). In animal models of DKD, enhanced mitochondrial fission in several types of renal cells has been reported to result in diminished energy production and accumulation of ROS, which further accelerate the progression of DKD (Sun et al. 2008; Ayanga et al. 2016). Not only mitochondrial fission has been recognized as a significant morphological sign of renal injury in DKD in numerous studies, but also excessive mitochondrial fusion participated in the pathogenesis of DKD (Kim and Lee 2021). Excessive fusion of mitochondria results in elongation and enlargement of mitochondria, leading to an increase in ROS production and a decrease in mitochondrial membrane potential (Woo et al. 2020; Yoon et al. 2006). In summary, imbalance of mitochondrial dynamics are closely related to the tissue damage of DKD by affecting mitochondrial function.

Mitochondrial dysfunction is altered by the production of superfluous ROS, accumulation of damaged mtDNA, and gradual dysfunction of the respiratory chain. It is known that excessive ROS production induced by high glucose is the predominant initiating damage mechanism in DKD (Lindblom et al. 2015). Overproduction of ROS may lead to oxidative stress and persistent harm to cellular constituents and glomerular podocyte, thereby leading to inflammation, interstitial fibrosis, and apoptosis in DKD (Fakhruddin et al. 2017; Badal, and Danesh 2014). Evidence suggests that the progressive accumulation of damaged mtDNA triggers an overproduction of ROS, subsequently causing mitochondrial dysfunction and subsequent DKD (Ježek et al. 2018). Simultaneously, mitochondrial damage leads to malfunction of the mitochondrial respiratory chain, leading to an inadequate intracellular ATP synthesis (Su et al. 2013), which further contributes to podocyte injury in DKD.

Under physiological conditions, cells can remove senescent or damaged mitochondria through mitophagy to maintain intracellular homeostasis (Galluzzi et al. 2017). Mitophagy disorders have also been confirmed to be involved in the pathogenesis of DKD (Zhang et al. 2021). The marker proteins of autophagy such as LC3, PINK1, Parkin, and Beclin1 are decreased while P62 is increased in renal cells and DKD models (Feng et al. 2018; Han et al. 2021; Wen et al. 2020; Sun et al. 2019; Guo et al. 2020; Palikaras et al. 2018). In kidney cells, the level of mitophagy in podocytes was higher compared tothat in renal tubular epithelial cells (Kitada et al. 2016; Hartleben et al. 2010). Enhanced mtophagy activity was demonstrated to have a renoprotective effect in rat models of DKD (Tagawa et al. 2016; Yang et al. 2019b). Supplement with MitoQ, a mitochondrial antioxidant, has been shown to protect from DKD by enhancing mitophagy levels (Xiao et al. 2017). Another study showed that inhibition of mitophagy in renal intrinsic cells significantly increased the number of damaged mitochondria whereas some factors such as PGRN, FoxO1, BNIP3 and FBW7 can alleviate renal inflammation and glomerular injury by activating the mTOR/Pink1/Parkin pathway to increase the level of mitophagy (Huang et al. 2016).

## The gut microbiota-microbial metabolites-mitochondrial axis in DKD

In 2011, Meijers and Evenepoel (2011) first put forward the theory of the gut-kidney axis. Later, Pahl and Vaziri (2015) proposed the “chronic kidney disease-colon axis” proposing that there may be mutual influence and interaction between human intestinal flora disorder and CKD progression. However, as the central medium of the ‘the gut-kidney axis’, the specific mechanism of gut microbiota between the intestine and the kidney is still unclear. There are still many unknown areas in the pathological process from intestinal flora disorder to chronic kidney damage that warrants further exploration. As previously summarized, although mitochondrial dysfunction and alteration of gut microbiota are both involved in the onset of DKD, it is not sure that whether the gut microbes send signals to host mitochondria to result in kidney damage.

**Fig. 2:**
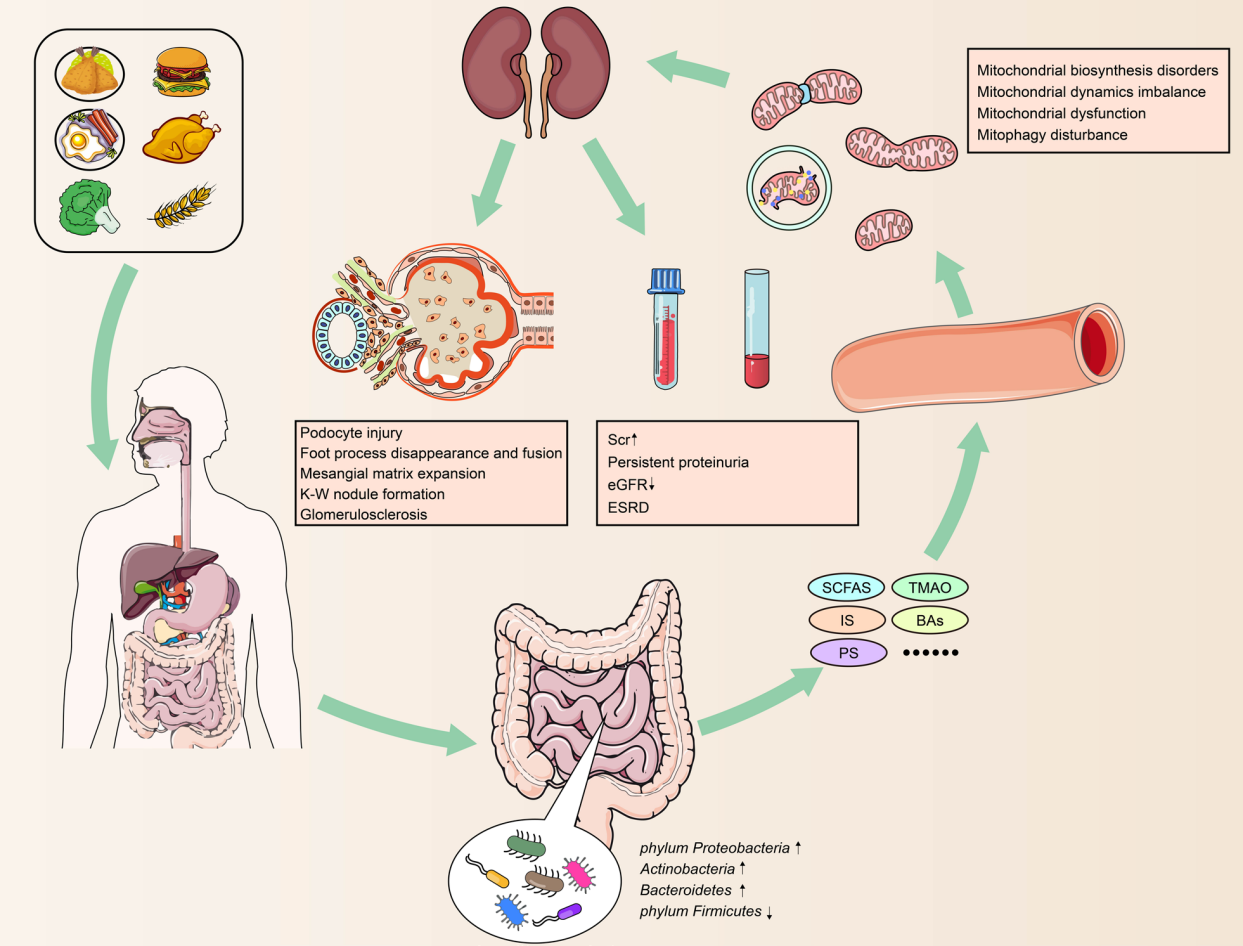
A potential biological function of the gut microbiota-microbial metabolites-mitochondria axis in the pathogenesis of DKD. In this hypothetical biological axis, with the deterioration of DM and digestion, degradation, and absorption of food by gut microbiota in the intestinal tract, the diversity and abundance of gut microbiota changed manifested by increased phylum Proteobacteria, Actinobacteria, Bacteroidetes and decreased phylum Firmicutes. Subsequently, various gut microbial metabolites are produced under the action of the gut microbiota and then released into the blood. These gut microbial metabolites, such as SCFAS, TMAO, PS and others, are transported to the kidney flowing the blood stream and interact with the mitochondria of kidney intrinsic cells. Metabolites of gut microbiota may lead to changes in mitochondrial function or disorder of mitochondrial quality control, thereby alleviating or accelerating the progression of DKD. *SCFAs* short-chain fatty acids, *TMAO* trimethylamine *N*-oxide, *IS* indoxyl sulfate, *PS* phenyl sulfate, *Bas* bile acids, *K–W* kimmelstiel–wilson, *Scr* serum creatinine, *eGFR* estimated glomerular filtration rate, *ESRD* end stage renal disease

More and more evidence have identified the critical function of microbial metabolites in the crosstalk of gut microbes and host mitochondria (Bajpai et al. 2018; Vezza et al. 2020). Therefore, based on the results of the current studies, we propose a potential biological axis including the gut microbiota-microbial metabolites-mitochondria in the pathogenesis of DKD. Therefore, we propose a potential biological axis including the gut microbiota-microbial metabolites-mitochondria in the pathogenesis of DKD based on the current studies (Fig. 2). In this hypothetical biological axis, the diversity and abundance of gut microbiota occurred in DM stage. With the deterioration of DM and degradation, digestion and absorption of food by gut microbiota in the intestinal tract, various gut microbial metabolites are produced under the action of the gut microbiota and then released into the blood. These gut microbial metabolites, such as SCFAS, TMAO, PS and others, are transported to the kidney flowing the blood stream and interact with the mitochondria of kidney intrinsic cells. Metabolites of gut microbiota may lead to changes in mitochondrial function or disorder of mitochondrial quality control, thereby alleviating or accelerating the progression of DKD. The emerging knowledge of the gut microbiota-microbial metabolites-mitochondrial axis may be of great importance to find new therapeutic options for DKD.

## Therapeutic strategies and future perspectives

The etiology of DKD involves multiple factors and pathways. In this review, we highlighted the potential mechanism of gut microbiota-microbial metabolites-mitochondrial axis in progression of DKD. As of yet, some therapeutic strategies including administration of probiotics, dietary intervention, and drugs have been used to delay acceleration of DKD. In the future, we should continue to explore the potential treatment for DKD based on the gut microbiota-microbial metabolites-mitochondrial axis.

Probiotics primarily aid in decreasing inflammation and oxidative stress, as well as slowing down the decline of kidney function in individuals with DKD (Vlachou et al. 2020). Several research studies have shown that probiotics can maintain intestinal permeability and enhance the integrity of cytoskeleton and tight junction proteins (Guo et al. 2017; Resta-Lenert 2003). He et al. reported that adding probiotics to the diet can improve the levels of blood glucose, lipids, and renal function in individuals with DKD by increasing the beneficial bacteria and reducing the bacteria released entheogenic endotoxins (He and Shi 2017). Taking soy milk containing Lactiplantibacillus plantarum A7 can significantly reduce Albuminuria, serum creatinine, IL-18 and Sialic acid in type 2 DKD patients (Abbasi et al. 2017). Jiang et al. illustrated that supplementation of probiotics (including bifidobacteria, Lactobacillus acidophilus, Streptococcus thermophilus) exerted beneficial effects on balancing the gut microbiota, reducing blood sugar and mAlb/Cr in DKD patients (Jiang et al. 2021). A systematic evaluation and meta-analysis revealed that probiotics can improve renal function, glucose and lipid metabolism disorders, oxidative stress and inflammation in DKD patients. This benefit is related to the intervention time, dosage, and consumption mode of probiotics (Dai et al. 2022).

Dietary fiber has been proved to modulate intestinal microbiota by promoting the enrichment of Prevotella and Bifidobacterium, which can produce SCFAs. In addition, dietary fiber can reduce not only the expression of profibrotic proteins but also genes encoding inflammatory cytokines and chemokines in diabetic kidneys (Li et al. 2020b). A potential non-pharmacological therapeutic strategy for DKD was fecal microbiota transplantation (FMT), which can modulate the gut microbiota through transplanting fecal bacteria obtained from fecal donors. Bastos et al. assessed the advantages of FMT on functional and morphological parameters in BTBR ob/ob mice, which mimic the pathogenesis of DKD in humans. The authors found that FMT inhibited body weight gain, decreased albuminuria and the levels of TNF-α in ileum and ascending colon (Bastos et al. 2022). Hu et al. (2020) proved that FMT could effectively reduce serum glycolic acid level in DN model rats and reduce cholesterol homeostasis disorder mediated by GPR43 activation, thereby diminishing renal tubulointerstitial damage and protecting the kidney.

Mitochondrial impairment has already been described in the process of DKD, thus, preventing mitochondrial damage and improving mitochondrial dysfunction could be a promising therapeutic approach for DKD. In fact, some studies have already demonstrated the feasibility of mitochondrial-targeted treatment for DKD. It has been reported that metformin can mitigate renal oxidative stress and tubulointerstitial fibrosis in DKD mice by enhancing the level of mitophagy through Pink1/Parkin pathway (Han et al. 2021). Ipragliflozin preserved renal tubular cells from high glucose or palmitate by up-regulating Mfn2 and OPA1, which are critical membrane GTPases for regulating mitochondrial biogenesis (Takagi et al. 2018). Empagliflozin, another SGLT2 inhibitor, has the ability to safeguard human renal proximal tubular cells against high-glucose damage by enhancing mitochondrial biogenesis, balancing fusion-fission protein, and triggering autophagy (Lee et al. 2019). Mitochondria-targeted antioxidant peptide SS-31can attenuate renal structural damages in DKD rat model and inhibit the production of ROS and mesangial cells apoptosis stimulated by high-glucose (Hou et al. 2016). Likewise, another study demonstrated that peptide SS31 can also alleviate renal tubulointerstitial injury through reducing fragmentation of mitochondria via inhibiting Drp1 and activating Mfn1 no matter in vivo and in vitro experiments (Yang et al. 2019a). Imeglimin, a new hypoglycemic drug targeting mitochondrial bioenergetics, was reported to enhance glucose tolerance and insulin sensitivity by protecting mitochondrial function against oxidative stress. Additionally, it can prevent the death of human endothelial cells caused by hyperglycemia through inhibiting the opening of permeability transition pores (PTP). Therefore, Imeglimin maybe a potential drug for treatment of microvascular complications in diabetic individuals (Vial et al. 2015; Detaille et al. 2016). Although many studies have confirmed that targeted mitochondrial therapy may delay the progress of DKD, they are all focused on animals and cells. In the future, more clinical studies are needed to clarify the prospect and effectiveness of mitochondria-targeted drugs for DKD.

As previously stated, the gut microbiota-microbial metabolites-mitochondrial axis is significant in the development of DKD, thereby, we propose a potential treatment approach based on the presence of such axis for DKD. SCFAs have been demonstrated to induce mitochondria genesis by activating AMP kinase (Cerdá et al. 2016). Acetate can be used as energy source by mitochondria (Lumeng and Davis 1973) and butyrate can stimulate mitochondrial biogenesis by inhibiting histone deacetylase (Galmozzi et al. 2013). Urolithin A was proved to enhance the oxidation capacity and mitochondrial function of skeletal muscle (Ryu et al. 2016). Therefore, the gut microbiota-microbial metabolites-mitochondrial axis may represent a promising perspective for exploring novel treatment for DKD. However, it is regrettable that no specific targeted drug has been found in clinical research that can regulate this axis. Supplementation of selected probiotics such as lactobacillus plantarum can increase the level of SCFA in colon (Molin 2001), which may exert potent effects in treatment for DKD by communicating with mitochondria. Whether we can infer that the benefits of dietary fiber, probiotics and prebiotics for treating DKD are related with intervening the gut microbiota-microbial metabolites-mitochondrial axis. In the future, more studies need to be implement to explore the specific mechanism of gut microbiota-microbial metabolites-mitochondrial axis in progression of DKD and application to new therapeutic strategies based on this axis.

## Conclusion

Although we have made a lot of efforts to explore the pathogenesis of DKD, we must be clear that we still lack effective treatment strategies to delay the continuous deterioration DKD. Thus it is necessary and urgent to find the new promising treatment schedule for DKD. With the development of metabonomics and metagenomics technology, the studies related to the gut-kidney axis have initiated a new promising perspective for exploring potential intervention strategies for DKD. Increasing evidence has demonstrated that mitochondrial dysfunction and alteration of gut microbiota and are both involved in the development of DKD. Microbial metabolites are supposed to be an important ‘bridge’ between gut microbiota and mitochondria in DKD. This article focused on the potential mechanism of gut microbiota-microbial metabolites-mitochondrial axis in progression of DKD. However, there are still some fundamental issues concerning the function of this biological axis in DKD unsolved so far. Firstly, which specific gut bacterium or microbial metabolites can specifically regulate mitochondrial function. Secondly, whether supplementation of different probiotics, prebiotics or microbial metabolites can slow down the advancement of DKD by alleviating mitochondrial damages. Finally, the target and molecular mechanism of microbial metabolites to improve or damage mitochondria are still unclear. Considering these questions, we anticipate more fundamental and clinical studies will be done to clarify the mechanism of gut microbiota-microbial metabolites-mitochondrial axis in DKD, which would shed great light on improving or delaying deterioration of DKD.

## Data Availability

Not applicable.

## Abbreviations

DKD: Diabetic kidney disease
ESRD: End-stage renal disease
DM: Diabetes mellitus
SGLT2: Sodium Glucose Cotransporter 2
CKD: Chronic kidney disease
ATP: Adenosine triphosphate
ROS: Reactive oxygen species
SCFA: Short-chain fatty acids
LPS: Lipopolysaccharides
H2S: Hydrogen sulfide
GF: Germ-free
IL-6: Interleukin-6
UACRs: Urinary albumin/urine creatinine ratios
GPR43: G-protein-coupled receptor 43
NF-κB: Nuclear factor kappa B
GPR109A: G-protein-coupled receptor 109A
TGF-β: Transforming growth factor-β
NaB: Sodium butyrate
HDAC: Histone deacetylase
NRF2: Nuclear factor erythroid 2-related factor 2
NRK-52E: Normal rat kidney tubular epithelial cells
GMCs: Glomerular mesangial cells
ICAM-1: Intercellular cell adhesion molecule-1
MCP-1: Monocyte chemotactic protein-1
IL-1β: Interleukin-1 β
TMAO: Trimethylamine *N*-oxide
NLRP3: Nucleotide-binding domain, leucine-rich-containing family, pyrin domain-containing-3 inflammasome
IL-18: Interleukin-18
FXR: Farnesoid X receptor
TGR5: G-protein-coupled BA receptor 1
UDCA: Ursodeoxycholic acid
TUDCA: Tauroursodeoxycholic acid
eNOS: Endothelial nitric oxide synthase
SOCS3: Suppressor of cytokine signaling 3
DDAH1: Dimethylarginine dimethylaminohydrolase 1
IS: Indoxyl sulfate
AhR: Aryl-hydrocarbon receptor
PS: Phenyl sulfate;
pCG: *p*-Cresyl glucuronide
PBUTs: Protein-bound uremic toxins
BCAAs: Branched-chain amino acids
eGFR: Estimated glomerular filtration rate
AD: Alzheimer’s disease
MS: Multiple sclerosis
PD: Parkinson’s disease
CVD: Cardiovascular diseases
K–W: Kimmelstiel–Wilson
HSV-1: Herpes simplex virus type 1
SIRT1: Silent information regulator 1
PGC-1α: Peroxisome proliferator-activated receptor-γ coactivator-1α
DRP1: Dynamic related protein 1
Fis1: Mitochondrial fission protein 1
MFF: Mitochondrial fission factor
OMM: Outer mitochondrial membrane
IMM: Inner mitochondrial membrane
MFN: Mitochondrial fusion protein
OPA1: Optic atrophy protein 1
